# Retrospective Comparison of 27-Gauge and 25-Gauge Microincision Vitrectomy Surgery with Silicone Oil for the Treatment of Primary Rhegmatogenous Retinal Detachment

**DOI:** 10.1155/2018/7535043

**Published:** 2018-09-16

**Authors:** Jie Li, Bo Zhao, Sanmei Liu, Fang Li, Wentao Dong, Jie Zhong

**Affiliations:** Department of Ophthalmology, Sichuan Academy of Medical Science & Sichuan Provincial People's Hospital, School of Medicine, University of Electronic Science and Technology of China, Chengdu, China

## Abstract

**Aim:**

To retrospectively compare the safety and effectiveness of 27-gauge (27G) microincision vitrectomy surgery (MIVS) with 25-guage (25G) MIVS for the treatment of primary rhegmatogenous retinal detachment (RRD) with silicone oil tamponade.

**Methods:**

Ninety-two patients with RRD who underwent MIVS from May 1, 2015, to June 30, 2017, were included in this study. Fifty-eight eyes underwent 25G MIVS and 34 eyes underwent 27G MIVS. We analyzed the characteristics of the patients, surgical time, main clinical outcomes, and rate of complications.

**Results:**

The mean surgical time was 56.7 ± 35.9 min for the 25G MIVS and 55.7 ± 36.1 min for the 27G MIVS, and there was no significant difference (*P*=0.894) between the two groups. The primary anatomical success rate after a single operation was 94.8% for 25G MIVS and 91.2% for 27G MIVS (*P*=0.666). Baseline and final visit best-corrected visual acuity (BCVA) were 1.9 ± 1.1 and 1.0 ± 0.8 in the 25G group, and 1.7 ± 1.0 and 1.1 ± 0.8 in the 27G group. Last visit BCVA increased significantly in both groups (*P* < 0.001). However, there were no significant differences in terms of visual improvement ratio (>0.2 logMAR) between the two groups (*P*=0.173). No severe intraoperative complication was observed. Iatrogenic retinal breaks occurred in 2 eyes (3.4%) in the 25G group and 1 eye (2.9%) in the 27G group during the peripheral vitreous base shaving. The transient ocular hypertension (>25 mmHg) within postoperative week 1 was 25.9% in the 25G group and 11.8% in the 27G group (*P*=0.120).

**Conclusions:**

This study found no significant anatomical or functional difference between 27G and 25G MIVS in the treatment of primary RRD. Therefore, 27G vitrectomy appears to be a safe and effective surgery for the treatment of primary RRD.

## 1. Introduction

Par plana vitrectomy (PPV), first introduced in 1971, has been widely used to treat posterior segment ocular diseases [1] and has evolved progressively driven by the desire for smaller instruments and greater functionality. More than a decade ago, 25- and 23-gauge microincision vitrectomy surgery (MIVS) instrument had dramatically simplified vitrectomy procedures [2–4]. In 2010, a 27G MIVS system was initially reported by Oshima et al. [5]. Recently, the feasibility of 27G MIVS has been demonstrated for various vitreoretinal diseases, including primary rhegmatogenous retinal detachment (RRD) [6–12]. However, there are only two prospective, comparative studies between 27G and 25G MIVS for RRD in a relatively small number (30 to 40 eyes) of patients so far [10, 13]. The purpose of this study was to retrospectively compare the safety and effectiveness of 27G MIVS with 25G MIVS for the treatment of primary RRD in 92 patients. This study would expand our current knowledge of the safety and effectiveness of the 27G MIVS.

## 2. Subjects and Methods

### 2.1. Study Design and Patients

We retrospectively reviewed 92 eyes of 92 patients with RRD who underwent 25G and 27G transconjunctival sutureless vitrectomy at the Sichuan Provincial People's Hospital (SPPH, Chengdu, China) by two surgeons (Dr. Zhong and Dr. Liu) from May 1, 2015 to June 30, 2017. This study has been proved by the Institutional Review Committee of SPPH and written informed consent was obtained from all patients before surgery. The procedures used conformed to the tenets of the Declaration of Helsinki. The inclusion criterion was primary RRD. The exclusion criteria were previous scleral buckling (SB) or vitrectomy, combined SB, trauma, proliferative vitreoretinopathy (PVR) of grade C, and significant ocular comorbidities such as uveitis, uncontrolled glaucoma, and severe or proliferative diabetic retinopathy. All patients underwent complete preoperative and postoperative ophthalmic examinations. The preoperative demographics of the patients are shown in Table 1.

### 2.2. Surgical Procedure

All surgeries were performed using a 25G or 27G MIVS (Constellation Vitrectomy system, Alcon Laboratories, Fort Worth, Texas, USA) and S88/OPMI Lumera T operation microscope with the RESIGHT wide-angle viewing operation system (Carl Zeiss Meditec AG, Germany). Retrobulbar anesthesia was induced with 2% lidocaine and conjunctival disinfection with povidone-iodine. An infusion cannula was inserted through the inferotemporal sclera followed by the oblique insertion of 2 cannulas through superotemporal and superonasal sites at a position 3.5 to 4 mm posterior to limbus. The infusion cannula was connected to the inferotemporal cannula. When phacoemulsification and intraocular lens implantation were combined, all prepositioned 25G cannulas kept closed until the vitrectomy began (27G cannulas were self-closed due to valved-cannula design). All cataract surgeries were performed through a 2.4 mm clear-corneal incision. The surgical parameters of vitrectomy were 7500 cycles per minute (cpm) for 27G vitrectomy and 5000 cpm for 25G vitrectomy and a vacuum of 0 to 650 mmHg. After the vitreal core was resected, we created posterior vitreous detachment (PVD) in case there was no spontaneous PVD presented. The peripheral vitreous was shaved as much as possible with scleral indentation. Perfluorocarbon liquid (PFCL, Fluoron) was used to stabilize retina if needed. Fluid-air exchange and subretinal fluid drainage were performed, and endophotocoagulation was performed around all retinal tears. As sulfur hexafluoride (SF6) or perfluoropropane (C3F8) was not commercially available in Mainland China since June, 2015, we used silicone oil (5000 centistokes, Zeiss) for intraocular tamponade in all cases. Silicon oil injection was required in 27G MIVS, the 27-gauge cannula at 10 O'clock was removed, and 25-gauge cannula was placed at the same sclerotomy site. Silicon oil was injected through 25-gauge oil injection syringe. Peripheral retina was examined before the removal of cannulas and no sclerotomy site suture was placed. Patients were strictly instructed to comply with a certain position depending on the location of retinal breaks.

### 2.3. Main Outcome Measurements

All patients had a complete ophthalmological examination at each clinic visit after the surgery. We analyzed data including age, patients' gender, laterality of the procedure, refractive error, preoperative intraocular pressure (IOP), preoperative and postoperative decimal best-corrected visual acuity (BCVA), extent of retinal detachment, initial anatomical success rate, final anatomical success rate, intraoperative and postoperative complications, and duration of silicone oil filling. Intraoperative data included operative time, complications, sclerotomy site leakage, and suture rate of sclerotomy wounds.

### 2.4. Statistical Analysis

All data were tabulated and organized with Microsoft Excel (Microsoft Corp., Redmond, WA). All statistical analyses were performed using IBM SPSS Statistics 24.0 (IBM Corporation, New York, USA). BCVA was measured using the Chinese E standard logarithmic visual acuity chart, and the decimal acuities were converted to the logarithm of the minimal angle of resolution (logMAR) units. For statistical analyses, counting finger (CF) vision was defined as 2.0 logMAR and hand movements (HM) were defined as 3.0 logMAR [13, 14]. Pearson's chi-squared test was used for intergroup comparisons of patients' gender, rate of combined surgery including cataract surgery, and rate of postoperative visual improvement. Independent t-test was used for intergroup comparisons of patients' age, preoperative IOP, and surgical time. Wilcoxon signed-rank test was used for intergroup comparisons of difference in preoperative and postoperative BCVA. *P* value < 0.05 was considered as statistically significant.

## 3. Results

### 3.1. Preoperative Characteristics

The mean age of the patients was 58.5 ± 13.3 years (range: 19–79 years) in the 25G MIVS group and 54.1 ± 12.5 years (range: 27–76 years) in the 27G MIVS group. There were 26 (44.8%) men and 32 (55.2%) women in the 25G group, and 11 (32.4%) men and 23 (67.6%) women in the 27G group. Baseline logMAR BCVA (mean ± SD) was 1.9 ± 1.1 (range: 3.0 to 0) in the 25G group and 1.7 ± 1.0 (range: 3.0 to 0.1) in the 27G group. Clinical data of the patients including lens status, choroidal detachment, proliferative vitreoretinopathy, and macular on/off status are shown in Table 1. Overall, there were no statistically significant differences in the preoperative characteristics of the patients between the 25G and 27G MIVS groups.

### 3.2. Surgical Time and Results

**Figure 1:**
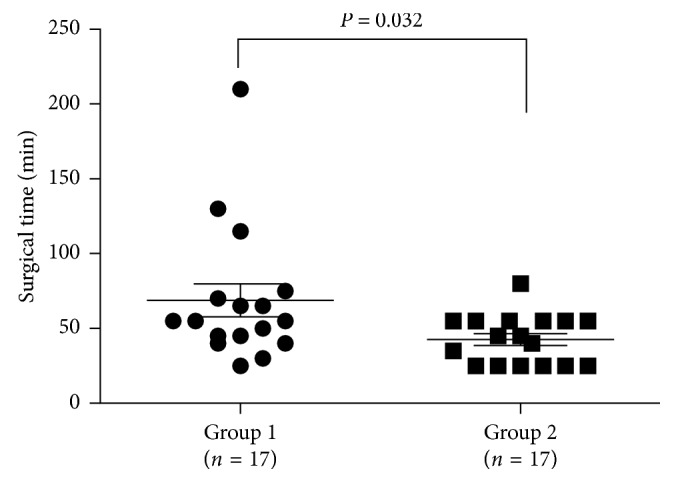
Surgical time used to treat RRD. Thirty-four eyes with RRD treated with 27G vitrectomy were equally divided into two groups. The mean operative time used to treat the first half (group 1, *n*=17) cases was 68.8 ± 45.4 min, which was significantly shorter than that (42.6 ± 16.3 min) used to treat the second half (group 2, *n*=17), *P*=0.032.

Nine (16.4%) phakic eyes in the 25G group and three (8.8%) phakic eyes in the 27G group had simultaneous phacoemulsification with intraocular lens implantation (*P*=0.489, Table 2). No complications occurred related to phacoemulsification such as posterior capsule rupture or zonular dialysis. The mean surgical time was 56.7 ± 35.9 min in the 25G group and 55.7 ± 36.1 min in the 27G group, and there was no significant difference (*P*=0.894) between the two groups. The mean surgical time of 27G vitrectomy was gradually shortened, when the surgeon's surgical technique was more and more adept. The mean operative time used to treat the second half of the RRD cases in the 27G group was 42.6 ± 16.3 min, which was significantly shorter than that of the first half (68.8 ± 45.4 min, *n*=17) (*P*=0.032, Figure 1). All retinal breaks received endolaser, and no external cryoapplication was applied. In both groups, silicone oil tamponade was used in all the eyes. After the removal of the microcannulas, no sclerotomy site was sutured in both groups. Silicone oil was removed at 3 to 6 months after the primary vitrectomy (range from 81 days to 299 days, median 125 days).

### 3.3. Anatomical Results

The primary anatomical success rate after a single operation was 94.8% and 91.2% in the 25G and 27G groups (*P*=0.666), respectively. In the 25G group, three (5.2%) eyes developed a recurrent retinal detachment during the follow-up period. In the 27G group, three (8.8%) cases of redetachment occurred during the follow-up period. The redetachment of the retina was due to PVR in four eyes (two eyes in the 25G group and two eyes in the 27G group) and new retinal breaks in two eyes (1 eye in the 25G group and 1 eyes in the 27G group). Three out of four eyes with PVR were reoperated using a novel two-port 27G vitrectomy technique without silicone oil removal as described in our previous study [15]. The other 3 cases were reoperated using conventional 25G PPV. Newly formed preretinal PVR membranes were peeled, subretinal fluid was drained, and all retinal breaks were sealed by endophotocoagulation with scleral depression. In the reoperated eyes, one eye underwent combined SB and one eye underwent silicon sponge placement. During the follow-up, one out of four PVR eyes developed a second recurrent retinal detachment and required relaxing retinotomy and long-term tamponade with SO.

#### 3.3.1. Changes of BCVA and IOP

**Figure 2:**
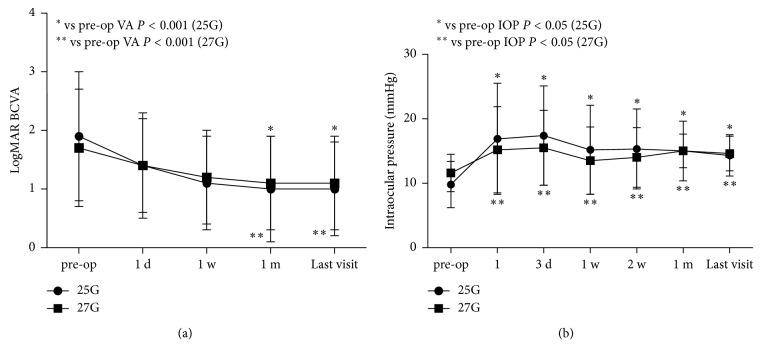
Time course of logMAR BCVA and IOP. (a) The mean logMAR BCVA at preoperative, postoperative day 1, week 1, month 1, and last visit was 1.9 ± 1.1, 1.4 ± 0.9, 1.1 ± 0.8, 1.0 ± 0.9, and 1.0 ± 0.8 in the 25G group, and 1.7 ± 1.0, 1.4 ± 0.8, 1.2 ± 0.8, 1.1 ± 0.8, and 1.1 ± 0.8 in the 27G group. When compared with the preoperative BCVA, postoperative BCVA at 1 month and the last visit increased significantly in both groups (*P* < 0.001). BCVA, best-corrected visual acuity, logMAR, logarithm of the minimal angle; (b) The mean IOP at baseline was 9.8 ± 3.6 mmHg in the 25G group and 11.6 ± 2.9 mmHg in the 27G group. At postoperative1 day, 3 day, 1 week, 2 weeks, 1 month, and last visit, IOP was 16.9 ± 8.6, 17.4 ± 7.7, 15.2 ± 6.9, 15.3 ± 6.2, 15.0 ± 4.6, and 14.3 ± 3.2 mmHg, in the 25G group, and 15.2 ± 6.7, 15.5 ± 5.8, 13.5 ±5.2, 14.0 ± 4.6, 15.0 ± 2.6, and 14.6 ± 2.7 mmHg in the 27G group. No significant differences in IOP between the two groups were observed during the follow-up. However, When compared with the preoperative IOP, postoperative IOP was significantly higher at each time point (*P* < 0.05) in both the 25G and 27G groups; IOP, intraocular pressure.

Baseline and final visit BCVA were 1.9 ± 1.1 and 1.0 ± 0.8 in 25G group and 1.7 ± 1.0 and 1.1 ± 0.8 in the 27G group, respectively (*P* < 0.001 for each intragroup comparison). Postoperative BCVA increased significantly in both groups at the one-month visit and the last visit postoperatively (*P* < 0.001, Figure 2(a)). No significant differences in terms of the visual improvement ratio at last visit (>0.2 logMAR) between the two groups (*P*=0.173) were found. The mean IOP at baseline was 9.8 ± 3.6 mmHg in the 25G group and 11.6 ± 2.9 mmHg in the 27G group. When compared with the preoperative IOP, postoperative IOP was significantly higher at each time point (*P* < 0.05, Figure 2(b)).

### 3.4. Intraoperative Complications

No severe intraoperative complication was observed. However, one eye in the 25G group (1.7%) and four eyes in the 27G group (11.8%) developed the slight posterior capsule opacification due to the extruding of the cutter probe on the posterior lens capsule. The capsular opacification facilitated the formation of postoperative cataract which required phacoemulsification with intraocular lens implantation during the secondary SO removal surgery. Iatrogenic retinal breaks (IRBs) occurred in two eyes in the 25G group (3.4%) and one eye in the 27G group (2.9%) during the peripheral vitreous base shaving. All IRBs were sealed carefully by intraoperative endolaser photocoagulation, and none of them resulted in postoperative rhegmatogenous retinal detachment.

### 3.5. Postoperative Complications

A comparison of postoperative complications between the 25G and 27G groups is shown in Table 3. There was no case of endophthalmitis, intraocular bleeding, or choroidal detachment noted in the follow-up period in both groups. There were two eyes in the 25G group (3.4%) and three eyes in 27G group (8.8%) that had subconjunctiva oil leakage during the follow-up period (*P*=0.355). Less than three eyes experienced severe hypotony (<6.5 mmHg) at each time point; there was no significant difference between the two groups. A few eyes encountered ocular hypertension (>25 mmHg) after both types of vitrectomy, but there was no significant difference between the two groups at each time point. The transient ocular hypertension within the first week postoperatively was 25.9% in 25G group, and 11.8% in 27G group (*P*=0.120). Most of the elevated IOP occurred within 1 month postoperatively (95.7% in 25G group, 100% in 27G group). Most eyes from both groups with an elevated IOP were treated with antihypertensive eye drops, and the IOPs returned to normal levels without glaucoma surgery. Four eyes with persistent elevated IOP (two eyes in the 25G group and two eyes in the 27G group, respectively) underwent antiglaucoma surgery.

## 4. Discussion

Although the transconjunctival sutureless 27G MIVS was first introduced in 2010, its surgical indications have been expanded from macular surgery to vitreoretinal diseases in the past few years. The safety and effectiveness of the 27G MIVS has been discussed and validated worldwide, the same as the evolution process of the previous 25G and 23G systems. Our aim was to further validate the safety and effectiveness of the 27G MIVS for the treatment of RRD. We focused on the surgical time, main clinical outcomes, visual improvement, and complications.

Operation effectiveness remains one of the most common concerns regarding 27G instrumentation, which has a smaller diameter probe (0.42 mm) than the 25G instrumentation (0.52 mm) [5]. Flow rate is the key factor for operation effectiveness. Theoretically, based on Poiseuille's law, a smaller vitrectomy probe would result in a greater flow resistance and slower flow rate. However, previous hydromechanics studies revealed that the 27G dual-pneumatic probes enable the control of duty cycles at an ultrahigh-speed cut rate (7500 cpm) and maintain an efficient vitreous flow rate [16, 17]. Mitsui et al. [6] found that the mean operative time for epiretinal membranes by 27G vitrectomy was 20.2 min, which was not significantly different from that of the 25G vitrectomy. In several other studies, the mean operative time of 27G vitrectomy for more complicated surgical indications ranged from 32.0 min to 36.3 min [7, 8, 10], which was comparable with the initial experiences with 25G equivalents [2, 18]. Similar to Romano's finding [10], we found no significant difference regarding the surgical time in treating RRD using 25G and 27G MIVS (*P*=0.894). When compared with the 25G group, the 27G group had a shorter surgical time, which might be due to the possibility that easier cases were chosen for 27G MIVS at the beginning of adoption of the 27G instrument. Furthermore, the surgical time for the 27G vitrectomy was gradually shortened when the surgeon became more skilled and got used to its flexibility. We found that the operative time used to treat the second half of the RRD cases in the 27G group was 42.6 ± 16.3 min, which was significantly shorter than that of the first half (Figure 1). Thus, although we cannot deny surgical effectiveness that could be affected by decreased flow rate within the smaller probe, our study suggested that surgical skill improvement and the high-speed cut rate of the 27G instrument could compensate for the reduced flow rate.

Similar to previous studies [10, 13, 19], last visit BCVA increased significantly in both the 25G and the 27G groups (*P* < 0.001). In terms of the visual improvement (>0.2 logMAR) ratio at last visit, there was no statistically significant differences between groups (*P*=0.177). Hence, our study demonstrated that the 27G MIVS was an effective way to treat RRD.

In addition, the primary anatomical success rate after a single operation was comparable in both groups (94.8% in the 25G group and 91.2% in the 27G group, *P*=0.666). The primary anatomical success rate using 27G MIVS was similar to earlier reports which ranged from 90% to 93% [10, 13]. These primary anatomical success rates using 27G MIVS are as good as, or even better than, that of the 25G MIVS which ranged from 74.0% to 95.45% [14, 20–22]. All patients in our study were treated with SO tamponade, and the primary anatomical success rates were similar to the previous study (93.5%) of patients with primary RRD operated on with vitrectomy and tamponated with silicone oil [23]. Meanwhile, SO tamponade may result in a lower rate of retinal displacement postoperatively [24]. Therefore, although definitive comparisons between studies are usually difficult, as they differ in many parameters, our results showed that the 27G PPV was as effective as the 25G in reattaching the retina after initial surgery.

Postoperative hypotony remains a major concern after sutureless vitrectomy, especially in 25G and 23G vitrectomy. With smaller wounds, 27G MIVS resulted in much lower rate of postoperative hypotony, ranging from 0 to 9.2% [6, 7, 10, 19]. Romano et al. used oblique incisions and displacement of the conjunctiva to reduce the severe hypotony postoperatively [10]. In our study, oblique incision was also implied and only 2.9% eyes experienced severe hypotony (<6.5 mmHg) in the 27G group. Since we had a higher rate of silicone oil tamponade than previous reports, this may contribute to lower the hypotony rate. On the contrary, in our study, the rates of transient ocular hypertension (>25 mmHg) in both groups (25.9% in the 25G group and 11.8% in the 27G group) within the first postoperative week were much higher than that of the previous reports with lower rate of SO tamponade, which ranged from 0 to 5% [7, 10, 13]. Similarly, Antoun et al. reported an incidence of ocular hypertension (defined as IOP > 21 mmHg) of 55% after vitrectomy surgery with SO tamponade [23]. This result suggested that a higher silicone oil infusion rate may result in a higher postoperative IOP.

Intra- and postoperative complications were similar in both the 27G and 25G groups. Postoperative endophthalmitis, intraocular bleeding, and choroidal detachment were not observed in both groups. IRBs and postoperative subconjunctival SO leakage rate were comparable between the 27G and 25G MIVS. It is worth noting that in the 27G group, there was a higher rate of posterior capsular injuries than that of the 25G group, although statistically significant difference was not reached (*P*=0.060). The capsular injuries were induced by cutter probe extruding on posterior capsular when performing the peripheral vitreous shaving. The reason why the 27G probe is more likely touch the posterior capsular may be because the 27G vitrectomy probe was more flexible and likely to be bent than the 25G instruments. Thus, we suggested that at the beginning of use of the 27G instruments for more challenging cases such as RRD, surgeons should keep in mind its property of flexibility, especially when performing the peripheral vitreous shaving. Nevertheless, this aspect did not affect the performance of the vitrectomy surgery. With the help of scleral indentation by a surgical assistant, there was no eye in the 27G group requiring conversion to larger-gauge instrumentation during peripheral vitreous shaving.

Similar to other surgeons, we found that the 27G instrumentation had several advantages [7, 13]. For example, using “3D” mode in constellation vitrectomy system, we could set a high cut rate with a low aspiration rate to reduce vitreoretinal traction from the probe tip, which would benefit peripheral shaving and possibly reduce iatrogenic retinal breaks. When performing the core vitrectomy, we can move to a more powerful aspiration rate to increase surgical efficiency. With its smaller diameter, the 27G probe can be more easily inserted into a tiny space such as subretinal membrane area. In addition, the current 27G vitrectomy probe featured a port placed 0.2 mm from the end of the probe. This feature could be used for BSS removal in the fluid-air exchange procedure and the removal of subretinal fluid. Therefore, the small, delicate, controlled, multifunctional 27G cutter saved time which was needed for the change of instruments. However, shorter effective work length of the 27G instrument restricts its application in the case with high myopia.

Our present study had several limitations. First, it was a retrospective, uncontrolled, noncomparative study from one center. Second, as the commercialized medical gas was recalled by China's State Food and Drug Administration in 2015 due to reported adverse events, we could not include gas tamponade data. Third, to perform silicone oil injection, we had to convert to a 25G oil injection syringe, which might also introduce potential bias and influence the data with respect to IOP, wound healing, and operative time. Nevertheless, our study suggested that the 27G vitrectomy with a high-speed cutting rate appeared to be as safe and effective as 25G vitrectomy in RRD surgery. Future randomized and controlled study with more patients is required to validate the advantages and disadvantage of 27G vitrectomy.

## Figures and Tables

**Table 1:** Characteristics of patients.

	25G	27G	*P* value
Number of eyes (*n*)	58	34	
Gender			0.239^†^
Male (*n*, %)	26 (44.8%)	11 (32.4%)	
Female (*n*, %)	32 (55.2%)	23 (67.6%)	
Age (years)	58.5 ± 13.3	54.1 ± 12.5	0.122^#^
Lens status			0.105^*∗*^
Phakic	48 (82.8%)	31 (91.2%)	
Pseudophakic	10 (17.2%)	2 (5.9%)	
Aphakic	0 (0.0%)	1 (2.9%)	
Preoperative BCVA, logMAR	1.9 ± 1.1	1.7 ± 1.0	0.322^‡^
Preoperative IOP (mmHg)	9.8 ± 3.6	11.6 ± 2.9	0.108^‡^
Preoperative choroidal detachment	11 (19.0%)	3 (8.8%)	0.240^*∗*^
PVR (*n*, %)	4 (6.9%)	1 (2.9%)	0.648^*∗*^
Macular-off (*n*, %)	49 (84.5%)	30 (88.2%)	0.761^†^

BCVA: best correct visual acuity; IOP: intraocular pressure; PVR: proliferative vitreoretinopathy. ^#^Independent *T* test; ^†^Pearson's chi-squared test; ^‡^Wilcoxon signed-rank test; ^*∗*^Fisher's exact test.

**Table 2:** Comparison of surgical procedures and results between groups.

	25G	27G	*P* value
Combine cataract surgery, *n* (%)	9 (16.4)	3 (8.8)	0.489^†^
Surgical time (min)	56.7 ± 35.9	55.7 ± 36.1	0.894^*∗*^
Postoperative IOP (mmHg)			
1 day	16.9 ± 8.6	15.2 ± 6.7	0.325^*∗*^
1 week	15.3 ± 6.2	14.0 ± 4.6	0.317^*∗*^
Last visit	14.3 ± 3.2	14.6 ± 2.7	0.600^*∗*^
Postoperative BCVA, LogMAR at last visit	1.0 ± 0.8	1.1 ± 0.8	0.780^‡^
Visual improvement	−1.0 ± 1.0	−0.6 ± 0.8	0.082^‡^
Visual improvement (>0.2 logMAR)	39 (67.2%)	18 (52.9%)	0.173^†^
Follow-up period (days, range)	185.7 ± 140.2	186.4 ± 126.7	0.982^*∗*^
Duration of silicone oil filling^#^	135.9.2 ± 39.8	132.6 ± 48.6	0.739^*∗*^

BCVA: best correct visual acuity; IOP: intraocular pressure. ^*∗*^Independent *T* test; ^†^Pearson's chi-squared test; ^‡^Wilcoxon signed-rank test; ^#^retinal redetachment excluded.

**Table 3:** Comparison of intraoperative and postoperative complications between groups.

	25G, *n* (%)				27G, *n* (%)	*P* value
Intraoperative complications						
Iatrogenic retinal breaks	2 (3.4)				1 (2.9)	1.000^‡^
Posterior capsule injuries	1 (1.7)				4 (11.8)	0.060^‡^
Intraocular bleeding	0				0	
Choroidal detachment	0				0	
Postoperative complications						
Endophthalmitis	0				0	
Redetachment	3 (5.2)				3 (8.8)	0.666^‡^
Subconjunctiva SO leakage	2 (3.4)				3 (8.8)	0.355^‡^
Severe hypotony (<6.5 mmHg)						
1 to 7 days postoperative	3 (5.2)				1 (2.9)	1.000^‡^
2 weeks to 1 month	1 (1.7)				0 (0.0)	1.000^‡^
Last visit	1 (1.7)				0 (0.0)	1.000^‡^
Elevated IOP (>25 mmHg)						
1 to 7 days postoperative	15 (25.9)				4 (11.8)	0.120^†^
2 weeks to 1 month	7 (12.1)				6 (17.6)	0.540^#^
Last visit	1 (1.7)				0 (0.0)	1.000^‡^

SO: silicone oil; IOP: intraocular pressure. ^†^Pearson's chi-squared test; ^‡^Fisher's exact test; ^#^chi-square test (continuity correction).

## Data Availability

The data used to support the findings of this study are available from the corresponding author upon request.
